# Associations Between Materialism, Gratitude, and Well-Being in Children of Overseas Filipino Workers

**DOI:** 10.5964/ejop.v14i3.1555

**Published:** 2018-08-31

**Authors:** Allan B. I. Bernardo, Roseann Tan-Mansukhani, Mary Angeline A. Daganzo

**Affiliations:** a Department of Psychology, University of Macau, Taipa, Macau; b Psychology Department, De La Salle University, Manila, Philippines; Department of Psychology, Webster University Geneva, Geneva, Switzerland; University of Neuchâtel, Neuchâtel, Switzerland

**Keywords:** overseas Filipino workers, materialism, gratitude, well-being, life satisfaction, self-esteem, children of migrants

## Abstract

Children left behind by parents who are overseas Filipino workers (OFW) benefit from parental migration because their financial status improves. However, OFW families might emphasize the economic benefits to compensate for their separation, which might lead to materialism among children left behind. Previous research indicates that materialism is associated with lower well-being. The theory is that materialism focuses attention on comparing one’s possessions to others, making one constantly dissatisfied and wanting more. Research also suggests that gratitude mediates this link, with the focus on acquiring more possessions that make one less grateful for current possessions. This study explores the links between materialism, gratitude, and well-being among 129 adolescent children of OFWs. The participants completed measures of materialism, gratitude, and well-being (life satisfaction, self-esteem, positive and negative affect). Results showed that gratitude mediated the negative relationship between materialism and well-being (and its positive relationship with negative affect). Children of OFWs who have strong materialist orientation seek well-being from possessions they do not have and might find it difficult to be grateful of their situation, contributing to lower well-being. The findings provide further evidence for the mediated relationship between materialism and well-being in a population that has not been previously studied in the related literature. The findings also point to two possible targets for psychosocial interventions for families and children of OFWs.

Recent official statistics of the Philippine government estimate that there are over 2.2 million Filipinos working overseas as temporary migrant workers, most of whom are deployed in countries in the Middle East, Asia, and Europe (Philippine Statistics Authority, 2017). Many of these overseas Filipino workers (henceforth, OFWs) are also parents, with one estimate indicating that in about 45% of families with OFW members, the OFW is one or both of the parents in that family (Edillon, 2008). There are no reliable data on the number of children left behind by these OFW parents, with estimates ranging from 3 to 6 million (Bryant, 2005) to 9 million (Parreñas, 2005). But the most conservative estimates suggest that around 10% of Filipino children have at least one parent who is an OFW (Bryant, 2005). For these children, there are material and economic benefits of their parents’ migration such as better education opportunities, access to leisure and recreation, and increase in material possessions (Aguilar, Peñalosa, Liwanag, Cruz, & Melendrez, 2009; ECMI/AOS, 2004; Yang, 2008).

But these material benefits might have some negative consequences as some studies assert that children of OFWs tend to view the “money equivalent” of labor migration (Reyes, 2008) and associate their parents with monetary benefits to compensate for their absence (Melgar & Borromeo, 2002). This view might lead to a materialistic orientation where the children are satisfied as long as the money from overseas parents comes regularly (Reyes, 2008; Scalabrini Migration Center, 2004). On the subject of materialism, previous research and theory have found a negative relation between materialism and various indicators of well-being (Kashdan & Breen, 2007; Kasser & Ahuvia, 2002) and that this relationship might be mediated by lower gratitude (Froh, Bono, & Emmons, 2010; Polak & McCullough, 2006). These studies are discussed in more detail later in the introduction, as the current study looks into the relationships between materialism, gratitude and well-being in children of OFWs. But first we briefly review some research on the well-being of Filipino children who are left behind by their OFW parents.

## Well-Being of Children of OFWs

The rise in the number of OFW parents has created many transnational families, where one or both parents reside in another country separate from their children. These OFW parents still try to do some “transnational parenting” while abroad, using various strategies and forms of communication technology to maintain emotional ties with their children (Parreñas, 2001). Some psychology studies have looked into the well-being of the children left behind by the OFWs in terms of their physical and mental health (Carandang, Sison, & Carandang, 2007; Scalabrini Migration Center, 2004), academic performance (Asis & Ruiz-Marave, 2013; Edillon, 2008), and risky behavior (Melgar & Borromeo, 2002). There are a few studies that have noted the difficulties experienced by children in families of OFW, despite attempts of their parents to maintain some form of transnational parenting. But as children of OFWs experience difficulties related to adjusting with the parents who leave (Asis, 2006; Carandang et al., 2007), these children also have an array of coping strategies for these difficulties (Asis, 2006; Battistella & Conaco, 1998). In terms of the children’s well-being, several studies have noted the experience of negative affect, such as feelings of loneliness and emptiness (Carandang et al., 2007; Melgar & Borromeo, 2002), and sadness (Battistella & Conaco, 1998; Scalabrini Migration Center, 2004). There are also some indications of psychological problems such as the experience of emotional distress (Battistella & Conaco, 1998), higher rates of dropping out of school, drug abuse, and other behavioral problems (Melgar & Borromeo, 2002).

One potential explanation that has been proposed for the psychological problems of some children of OFWs relates to the possibility that some children of OFWs may view their relationship with their OFW parent primarily in financial and material terms (Añonuevo & Sopeña, 2002; Reyes, 2008). There has been more extensive work done understanding how transnational parenting has given rise to the *commodification of love* as a coping strategy among OFW parents (Parreñas, 2001, 2005). Research suggests that OFW parents, and OFW mothers in particular, tend to cope with the emotional strains of transnational parenting by commodifying love (Parreñas, 2001); they overcompensate for their absence by giving their children all the money and material goods that the children want. On the part of the children, studies have noted how some children of OFWs tend to focus on the “money equivalent” of their parents’ migration (Reyes, 2008) and associate their parents with monetary benefits to compensate for their absence (Melgar & Borromeo, 2002). Other researchers have suggested that these children develop a strong consumerist and materialistic orientation, where the children are satisfied as long as the money from overseas parents comes regularly (Reyes, 2008; Scalabrini Migration Center, 2004). We note that these studies did not actually measure endorsement of materialistic values, and these proposals that this form of parent-child relationship gives rise to materialistic values in children of OFWs were derived from intensive interviews of samples of children of OFWs. But there are studies in other countries that show how parents’ use of material goods in parenting leads to materialism in the children (Richins & Chaplin, 2015). In the next section we review some studies on the negative consequences of such materialism on well-being.

## Materialism, Well-Being, and Gratitude

Materialism refers to “a strong desire for wealth and physical possessions” (Kashdan & Breen, 2007, p. 522) and has been known to decrease well-being (Kashdan & Breen, 2007; Kasser & Ahuvia, 2002; Polak & McCullough, 2006). Although much of the empirical research on the negative consequences of materialism has been done with adults, research also indicates that materialistic youth tend to have decreased well-being and mental health (Kasser & Ahuvia, 2002), show lower levels of psychological adjustment and social functioning (Kasser & Ryan, 1996), and are less socially integrated in the community (Froh et al., 2010). Materialism has also been consistently associated with higher levels of anxiety and unhappiness (Kasser & Ahuvia, 2002) and lower affective well-being (Christopher, Saliba, & Deadmarsh, 2009). More materialistic children and adolescents also show less interest in school (Goldberg, Gorn, Peracchio, & Bamossy, 2003), have poorer motivation for learning in school (King & Datu, 2017; Ku, Dittmar, & Banerjee, 2012) and actually show poorer academic performance (King & Datu, 2017; Ku, Dittmar, & Banerjee, 2014). Such negative consequences of materialism on well-being have been replicated in different cultural contexts (Karabati & Cemalcilar, 2010; Ryan et al., 1999; Stevens, Constantinescu, & Butucescu, 2011), including the Philippines (King & Datu, 2017) and other Asian countries (Choong, Ong, & Moschis, 2013; Kasser & Ahuvia, 2002; Ku et al., 2012, 2014).

Why is materialism negatively associated with well-being? One typical explanation relates to the notion that the pursuit of materialistic values necessarily relates to excessive interpersonal comparison of one’s possessions relative to other persons (Lyubomirsky & Ross, 1997) and so one continuously feels dissatisfied with one’s wealth and status relative to other persons (Ordabayeva & Chandon, 2011). This continuous feeling of dissatisfaction also arises from the adaptation of the hedonic feelings relative to one’s possessions, as one will always see better things available (Lyubomirsky, 2011). In this way, materialistic thinking is likely to result in negative emotions and low self-worth (Kernis, Brown, & Brody, 2000). More importantly, materialistic values draw a person’s focus away from behaviors that satisfy basic psychological needs (e.g., relatedness, autonomy, competence), which are important sources of well-being (Ryan & Deci, 2000; Sheldon, Elliot, Kim, & Kasser, 2001).

Developmental studies suggest that materialism surges during late childhood and early adolescence, and then declines through late adolescence and most of adulthood (Chaplin & Roedder John, 2007), but that levels of materialism in late adolescence and early adulthood are strongly associated with levels of materialism in late adulthood (Jaspers & Pieters, 2016). The shifting levels of materialism during adolescence seems to be associated with the formation of identity and the sense of self (Chaplin & Roedder John, 2005), wherein material possessions are seen to express aspects of themselves, their enjoyment and social ties (Kamptner, 1991). Empirical studies show how later levels of materialism is associated with psychological needs’ satisfaction (Wang, Liu, Jiang, & Song, 2017), coping with loneliness (Gentina, Shrum, & Lowrey, 2016), and earlier life satisfaction (Ku, 2015).

As noted earlier, the relation between materialism and lower well-being has been established across different cultural contexts, and as such it seems to be a “pan-cultural phenomenon.” However, there are specific contextual factors that seem to play a role in the development of materialism and that could also be seen as pointing to specific cultural sources of materialism. For example, for adolescents, parenting and family structure (Chaplin & Roedder John, 2010; Roberts, Manolis, & Tanner, 2006), and especially having materialistic parents (Kasser, Ryan, Zax, & Sameroff, 1995) predict materialism. Less advantageous socioeconomic circumstances also seem to be associated with higher levels of materialism (Chaplin, Hill, & Roedder John, 2014; Kasser et al., 1995), which also explains variations in levels of materialism across generations which face different socioeconomic conditions (Twenge & Kasser, 2013). Adolescents also seem to be particularly influenced by marketing promotions and media exposure to consumerism values (Gu, Hung, & Tse, 2005).

Many psychological processes have been proposed as mechanisms for the negative relationship between materialism and well-being. Some researchers have proposed that gratitude might play a role in the negative relationship between materialism and well-being (Tsang, Carpenter, Roberts, Frisch, & Carlisle, 2014). Gratitude is defined as the “recognition and acknowledgement and appreciation of an altruistic act” (Emmons, 2004, p. 9). It involves the understanding of the fact that one has gained a positive outcome and that an external agent is responsible for the outcome (McCullough, Kilpatrick, Emmons, & Larson, 2001). In adult and youth samples, gratitude is consistently associated with various indices of well-being (Wood, Froh, & Geraghty, 2010), such as life satisfaction (Lavy & Littman-Ovadia, 2011; Toepfer, Cichy, & Peters, 2012), positive affect (Froh, Kashdan, Ozimkowski, & Miller, 2009), decreased negative affect (Froh, Sefick, & Emmons, 2008), decreased depression (Emmons & McCullough, 2003; Toepfer et al., 2012), higher self-esteem (Rash, Matsuba, & Prkachin, 2011), among others. These positive outcomes of gratitude have also been observed with Filipino adolescent samples (Datu, 2014; Datu & Mateo, 2015; Valdez, Yang, & Datu, 2017).

Materialism has been associated with lower levels of gratitude (Froh et al., 2010; McCullough, Emmons, & Tsang, 2002; Polak & McCullough, 2006). One study has shown that gratitude is one of the mediators of the negative relationship between materialism and well-being (Tsang et al., 2014). The study suggests that individuals who show high materialism are seeking satisfaction in material possessions that they do not have; and because they keep comparing their possessions to other people, they constantly think about what they do not have in comparison (Lyubomirsky & Ross, 1997). This focus of one’s attention on what one does not have, draws away attention from what they have presently; the feeling fostered is one of continuous dissatisfaction with one’s current possessions (Ordabayeva & Chandon, 2011) to the detriment of feeling gratitude for what they have presently (Tsang et al., 2014). This explanation might apply in the case of children of OFWs who may be expecting constant material and financial gifts from their OFW parents, and who might define their life satisfaction with reference to how these material goals are met.

## The Current Study

In this study, we investigate whether materialism in children of OFWs also relates to lower well-being, and whether this relationship is mediated by lower levels of gratitude. Note that we are not assuming that children of OFWs all show high levels of materialism, and instead, we assume that materialism would be normally distributed among the children of OFWs. But we hypothesize that those children of OFWs who report higher levels of materialism are also likely to report lower levels of gratitude and well-being. To test this hypothesis, we look at four measures of well-being: life satisfaction, self-esteem, positive affect, and negative affect, the last being a measure of lower well-being. Life satisfaction is the most frequently used measure of well-being according to a meta-analysis of studies on materialism, although its associations with materialism is not as strong as the others (Dittmar, Bond, Hurst, & Kasser, 2014). Measures of positive self-appraisal or self-esteem were studied less frequently, but were found to have stronger negative relationships with materialism, perhaps because of the role of the sense-of-self in the development of materialism (Chaplin & Roedder John, 2005). Both positive and negative affect are also frequently used as measurements of well-being in materialism research, and both measures of affective well-being having strong and consistent associations with materialism in the meta-analysis (Dittmar et al., 2014). In summary, we used a general measure of subjective well-being (life satisfaction), a measure of positive self-worth (self-esteem), and two measures of affective well-being (positive and negative affect), all of which are frequently used in materialism and well-being research, and are found to have consistent associations with materialism.

We summarize our hypotheses, as follows:

1. materialism will be negatively associated with life satisfaction, self-esteem and positive affect, but positively associated with negative affect;
2. materialism will be negatively associated with gratitude;
3. gratitude will be positively associated with life satisfaction, self-esteem and positive affect, but negatively associated with negative affect; and
4. gratitude will mediate each of the relationships between materialism and the four well-being measures.

## Method

### Participants

Participants were 129 undergraduates from two private universities in the Philippines who reported that at least one of their parents was working abroad. Their ages ranged from 18 to 20 years (*M* = 18.68; *SD* = 1.46) and most were female (75.2%). Most (58%) reported that their father was working abroad, while 22% had their mother working overseas, and 20% had both parents working overseas. All participants were recruited to participate in the study through their teachers, and the invitation included information on the nature of the study, specifying that participation was voluntary, confidential, and involved no harm, and other pertinent ethics information.^i^ Only those who provided their informed consent, participated in the questionnaire. Participants did not receive payment or get class credit for participating.

### Instruments

All the psychological scales used in the study are typically used in similar studies. The measures and scores for measuring materialism and gratitude are the most commonly used in related studies, and all measures of well-being are also among the most commonly used scales in psychology of well-being research.

#### Aspiration Index

To measure the participants’ materialism, the 22-item Aspiration Index (Kasser & Ryan, 1996) was used to measure intrinsic and extrinsic aspirations; intrinsic aspirations are goals congruent with basic psychological needs (e.g., self-acceptance, affiliation, community feeling) and extrinsic aspirations concern obtaining some material or social rewards (e.g., financial success) that are usually means to some other end. The scale items described different goals that a person may aspire to, and the participants were asked to rate how important each of these was for them using a scale of 1 (*not at all*) to 5 (*very important*). For intrinsic aspirations (sample item: “You will have good friends that you can count on”), Cronbach α = .87, and for extrinsic aspirations (sample item: “You will buy things just because you want them”), Cronbach α = .68. Materialism was measured by computing the difference between the mean score for the extrinsic and intrinsic aspirations. The actual scores ranged from -2.17 to 2.40, with a higher score indicating greater materialism.

#### Gratitude Questionnaire–6 (GQ-6)

The shortest version of the gratitude scale developed by McCullough et al. (2002) was used. For each of the six items (e.g., “If I had to list everything that I felt grateful for, it would be a very long list.”) participants responded using a scale from 1 (*strongly disagree*) to 7 (*strongly agree*). The scale had adequate internal consistency for the current sample (Cronbach α = .66); although α is below conventional criterion, the scale was still used because previous studies using the scale with similar Filipino samples have shown acceptable internal consistency (e.g., Datu, 2014).

#### Satisfaction With Life Scale (SWLS)

The SWLS (Diener, Emmons, Larsen, & Griffin, 1985) was used to assess general well-being. The scale had five items (e.g., “The conditions of my life are excellent”) to which the participants indicated their agreement using a scale from 1 (*strongly disagree*) to 7 (*strongly agree*). The scale had good internal consistency for the current sample (α = .83).

#### Self-Esteem Scale

Rosenberg’s (1965) 10-item scale (e.g., “I take a positive attitude towards myself”) was used. Participants indicated their agreement using a scale from 1 (*strongly disagree*) to 4 (*strongly agree*). The scale had good internal consistency for the current sample (α = .85).

#### Positive and Negative Affect Schedule (PANAS)

The scale (Watson, Clark, & Tellegen, 1988) consisted of 20 words that referred to 10 positive (e.g., excited, attentive) and 10 negative (e.g., hostile, ashamed) emotions. Participants reported the extent of how they felt each emotion in the past week using a scale from 1 (*very slightly or not at all*) to 5 (*extremely*). Both scales had good internal consistency for the current sample (positive affect, α = .88; negative affect, α = .88).

## Results

The descriptive statistics for all variables are presented in Table 1. The correlations all support the first three hypotheses. In particular, materialism was negatively associated with gratitude and well-being (but positively with negative affect), while gratitude was positively associated with well-being (but negatively with negative affect).

**Table 1:** Descriptive Statistics for All Main Variables

Variable	*M*	*SD*	Correlations (*r*)				
			2	3	4	5	6
1. Materialism	-0.05	0.71	-.32**	-.20*	-.19*	-.32**	.20*
2. Gratitude	5.87	0.74	–	.27**	.43**	.32**	-.30**
3. Life satisfaction	5.29	1.02		–	.57**	.50**	-.18*
4. Self-esteem	3.01	0.51			–	.55**	-.41**
5. Positive affect	3.94	0.69				–	-.15
6. Negative affect	2.71	0.76					–

*p < .05. **p < .01.

Prior to the mediation analyses, we conducted test of tolerance and variance inflation factor (VIF) to ensure that there was no multicollinearity (i.e., high correlations between two or more of the variables) in the hypothesized mediation models. All tolerance scores were from .88 to .99 and all VIFs were from 1.01 to 1.14, thus ensuring no multicollinearity.

To test each hypothesized mediated relationship between materialism and each of the four measures of well-being we used the PROCESS macro for SPSS, which is a computational tool for conducting mediation and conditional process analysis with observed variables (Hayes, 2013). To estimate the parameters of each equation in the path analysis of the mediated model, PROCESS uses ordinary least squares regression. Unlike in the classical regression framework for mediation (Baron & Kenny, 1986; Hayes, 2009), each equation is estimated separately, which means that the estimation of the regression parameters in one equation does not have any effect on the estimation of the parameters in any of the other equations defining the model. Similar to SEM, PROCESS produces estimates of the indirect effect (which is not estimated in the classic regression framework of Baron & Kenny, 1986), and does not require separate tests (e.g., Sobel or Aroian tests) to assess the significance of the mediation effect. But unlike SEM, which requires normal sampling distributions and large sample sizes, PROCESS can be used with smaller sample sizes that may have irregular sampling distributions because the estimates of the indirect effects are based on bootstrapping methods (Hayes, 2013; Hayes, Montoya, & Rockwood, 2017). As bootstrap confidence intervals consider the irregularity of the sampling distribution, the inferences are more likely to be accurate and the test of a hypothesis has higher power compared to when normal theory approach is used (Hayes, 2013), like when doing SEM.

**Figure 1:**
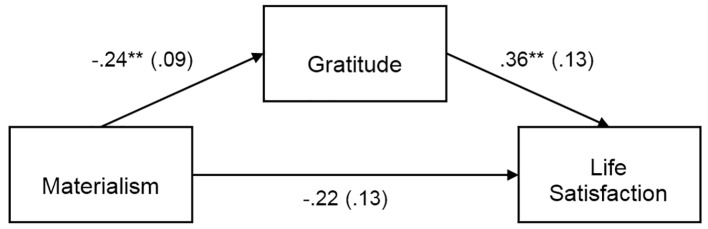
Materialism indirectly predicts life satisfaction through its relationship with gratitude, *b* = -.08, *SE* = .05, 95% CI [-.22, -.02]. Unstandardized coefficients shown with standard error in parentheses. **p < .01.

**Figure 2:**
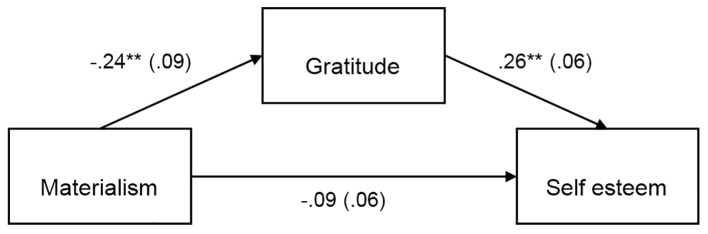
Materialism indirectly predicts self esteem through its relationship with gratitude, *b* = -.06, *SE* = .03, 95% CI [-.14, -.02]. Unstandardized coefficients shown with standard error in parentheses. **p < .01.

**Figure 3:**
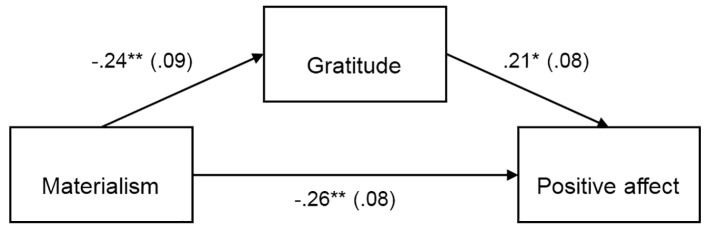
Materialism indirectly predicts positive affect through its relationship with gratitude, *b* = -.05, *SE* = .03, 95% CI [-.13, -.01]. Unstandardized coefficients shown with standard error in parentheses. *p < .05. **p < .01.

**Figure 4:**
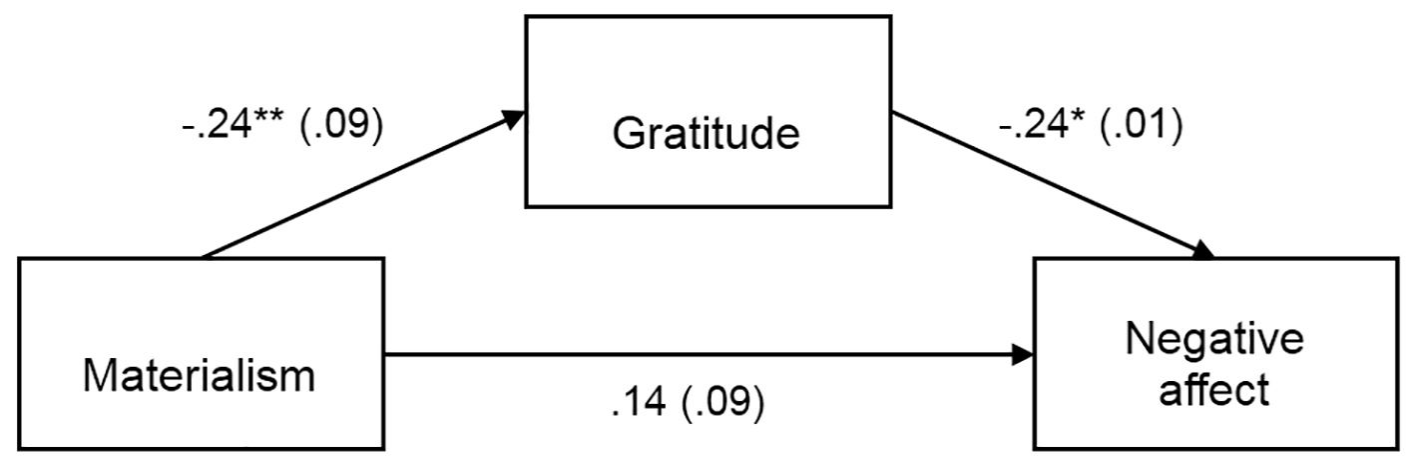
Materialism indirectly predicts negative affect through its relationship with gratitude, *b* = .06, *SE* = .04, 95% CI [.01, .16]. Unstandardized coefficients shown with standard error in parentheses. *p < .05. **p < .01.

Separate PROCESS mediation analyses were conducted to test the indirect effect of materialism on life satisfaction, self-esteem, positive affect and negative affect with gratitude as mediator. The results of the PROCESS analyses are summarized in Figures 1 to 4, which show the path coefficients and their respective 95% bias-corrected confidence intervals.

The direct effect of materialism on life satisfaction, self-esteem, and negative affect were not significant, as shown in Figures 1, 2 and 4. But as shown in Figure 3, there was a significant direct (negative) effect of materialism on positive affect. But the indirect effects as indicated in the bootstrap confidence intervals in all four figures were all significant, indicating a mediated relationship between materialism and each of the four measures of well-being. Thus, even if the direct effect was significant in Figure 3, the hypothesized mediated relationship between materialism and well-being, with gratitude as mediator was supported in all four measures of well-being.

## Discussion

The study aimed to investigate the relationship between materialism and well-being and the mediating role of gratitude in this relationship, in the case of children of OFWs. Consistent with much of the previous literature (Kashdan & Breen, 2007; Kasser & Ahuvia, 2002), we found support for our first hypothesis, and found that materialism was negatively correlated with the children’s well-being (and positively correlated with lower well-being, i.e., negative affect). The path analysis, however, showed that in three measures of well-being the direct relationship between materialism and well-being was not significant, which indicates that the hypothesized first relationship needs to be clarified to state that the negative relationship between materialism and well-being may be an indirect or mediated relationship. This clarification is expressed in the other hypotheses, which are supported by the correlations and path analyses; that is, materialism was negatively associated with gratitude, and gratitude was positively associated with well-being. Most important and consistent with earlier proposals (Froh et al., 2010; Polak & McCullough, 2006), gratitude mediated each of the relationships between materialism and well-being.

In earlier studies, Tsang et al. (2014) suggest that people with high levels of materialism look for happiness and satisfaction from the material possessions they do not have, which “impairs the ability to be grateful for what one has now” (p. 63). Indeed, materialists do tend to compare their material possessions to those that other people have, making them always think of what they do not have compared to others (Lyubomirsky & Ross, 1997) making them dissatisfied with whatever materials possessions they might already have (Ordabayeva & Chandon, 2011), thus enfeebling whatever feelings of gratitude one might have for the current possessions (Tsang et al., 2014). Our results suggest that these theoretical principles might also apply to children of OFWs. Note that the mean materialism score was slightly below zero, which suggests that on the average the children of OFWs in our sample are not high in materialism, contradicting what has been proposed by some other researchers (e.g., Reyes, 2008) who suggest that children of OFWs might develop strong consumerist and materialist orientations. Instead, what we see is a range in the materialism scores, and applying the principles discussed earlier, it seems that those children of OFWs who are high in materialism seem to be less inclined to be grateful of their current situation, indirectly contributing to their lower well-being. This pattern is consistent with results of Tsang et al. (2014), and extends their findings to a new type of sample (children of OFWs) and to a wider range of measures of well-being.

We did not hypothesize any differences in the relationships between the various measures of well-being and materialism, but it seems that the direct negative relationship of well-being is on the positive affect experienced by the children of OFWs, which is not observed with the three other well-being measures. This result is consistent with a meta-analysis of research on materialism (Dittmar et al., 2014), which indicated the strongest relationships between materialism and positive affect compared to all other positive well-being measures (including self-worth and life satisfaction). The association between gratitude and well-being seemed to be stronger with the more specific affect and self-esteem measures and weakest with life satisfaction, which is the most general measure of subjective well-being (i.e., sources of life satisfaction may vary considerably across individuals).

We should underscore that the negative relationship between materialism and well-being and the mediating role of gratitude in this relationship is not unique or specific to the population of children of OFWs, and is actually observed in other samples (Tsang et al., 2014). Other researchers have suggested that the negative effect of materialism on well-being can be explained in terms of how materialism might lead to the deprioritizing of other basic psychological needs (i.e., relatedness, autonomy, and competence) that are most important in experiencing well-being (Kasser, 2016; Kasser & Ryan, 1996). Moreover, the pursuit of materialist goals makes it more difficult to be grateful for their current possessions and other psychological needs, as these may be perceived as being in conflict with their material pursuits (Burroughs & Rindfleisch, 2002; Tsang et al., 2014).

But the negative relationship between materialism and well-being, and especially the mediating role of gratitude may have different nuances of meaning if we concretize this in the family experiences of children of OFWs. Popular media and social discourses about OFWs in the Philippines typically cast the OFW as heroes of the nation, who sacrifice their own well-being and endure separation from their families to contribute to the nation’s economy (Encinas-Franco, 2013; Rodriguez, 2002), but children of OFWs may construe their OFW parents’ actions in different ways. Some children view their OFW parent’s migration as a means of helping their family improve their financial condition, while others see it as abandonment (Battistella & Conaco, 1998; Reyes, 2008) even as they may be receiving the material benefits of their parent’s work overseas. The results of the study suggest that children of OFWs who hold more materialistic values may be less likely to appreciate and experience gratitude in relation to their OFW parents’ migration. Interestingly, research has shown how OFW mothers, in particular, give their children whatever material possessions they request as a way of overcompensating for their absence (Parreñas, 2001, 2005); in a sense, satisfying the material needs, but not the relational needs. It might be tempting to assume that this *commodification of love* may contribute to materialism in some of their children. However, the results of the study cannot address that assumption, even as there is research which indicates that parenting plays a very important role in shaping materialism in children (Flouri, 2004; Goldberg et al., 2003). Future research should explore further whether and how transnational parenting relates to OFW children’s materialism. Further research should also investigate the role of the parents and guardians of the children, as well as other factors that may contribute to the development of materialism in individuals.

Related to the last point, we earlier noted that on the average, the children of OFWs in our sample did not report high levels of materialism. But we underscore that our study involved a small sample, which is not representative of the growing population of such children in the Philippines, and thus, not representative of the range of transnational parenting experiences and expectations of this population. There are several sociological studies that point to the use of materialism in some OFWs transnational parenting (Parreñas, 2001, 2005) and to how children perceive their relationships with their parents in financial terms (Melgar & Borromeo, 2002; Scalabrini Migration Center, 2004), which has led some others to suggest that children of OFWs might develop stronger materialist orientations (Reyes, 2008). Further research is needed with regard to the link between parenting practices, children’s experiences and expectations of their parents, and materialism in order to further explore this claim, particularly as our results point to materialisms’ negative relationship with the children’s well-being.

Previous research on children left behind by labor migration focused on the psychosocial consequences on well-being, and economic benefits of remittances, and only a few studies have emphasized the inner resources of children to deal with the challenges faced by OFW families (e.g., Asis, 2006). Research among other youth populations have suggested that character strengths like gratitude offer a means for fostering positive youth development (Froh, Miller, & Snyder, 2007; Froh et al., 2009). As such, it may be important to focus on the development of gratitude in children of OFWs, particularly as one study has suggested that Filipino youth may prioritize value types associated with materialism (i.e., power and stimulation) to a higher degree compared to “pan-cultural” norms (Bernardo, Clemente, & Liem, 2014).

While traditional Filipino child-rearing practices placed the responsibility of values formation of children squarely on the shoulders of parents, the non-traditional OFW family structure has shifted the responsibility of care, and by extension, the socialization of values, on parent surrogates, whether members of the family or the community. Programs or activities for enhancing gratitude among the youth may be conducted in schools (Froh et al., 2007), particularly those that focus on the development of socially oriented gratitude (Caleon et al., 2017). In this regard, there have been attempts to develop interventions programs that seek to provide information, assistance, and support for families of OFWs. Most of these programs take the form of psycho-educational and psychosocial support activities that target the children, and also the spouse and other family members of the OFW. Although there has been no systematic or comprehensive documentation of such programs, those programs that have a psychosocial focus on children seem to focus on facilitating communication, values formation, and coping skills to help address the negative affect they experience (Tarroja & Fernando, 2013). Tarroja and Fernando (2013) noted that some schools provided psychological counseling programs for children of OFWs but observed that these interventions are not programmatic; and there has been no explicit articulation of the potential problems associated with changes in the financial status of the children and their families, even as there is some suggestion that some children of OFWs may view their relationship with their OFW parent primarily in financial and/or material terms (Añonuevo & Sopeña, 2002; Reyes, 2008). The current findings suggest that psychological programs conducted for children of OFWs may be correctly focusing on values formation, even as these programs actually aim to address adjustment and interpersonal difficulties in the OFW family (Tarroja & Fernando, 2013). The results indicate that some values that contribute to the well-being of OFW children may actually be diminished in the experience of OFW children who are more materialistic. Thus, it may be worth considering helping children of OFWs to develop stronger values related to gratitude, but also to nurture those value orientations that are the opposite of materialism, such as valuing competence, relationships, and contributing to society, which are already distinct value types in adolescence (Liem, Martin, Nair, Bernardo, & Prasetya, 2011).

We should note that although the mediated model seems to suggest some causal ordering of the key variables, our results are primarily correlations based on cross-sectional self-report data. Future research should try to establish a clearer basis for understanding how these values relate to well-being, as these might provide better guides for psychological programs intended to improve and sustain the well-being of children of OFWs. We also acknowledge that the children of OFWs that we studied are older adolescents and young adults living in an environment that provides exposure to consumerist attitudes, and so the relationships we describe among the variables are likely to have been shaped by many other processes outside the OFW family structure. Moreover, as noted in the introduction, materialism is higher among older children and younger adolescents (Chaplin & Roedder John, 2007), compared to older adolescents who comprise the current study sample. There might be developmental differences associated with materialism among younger and older children of OFWs. And it would be interesting to understand whether younger children of OFWs show the same or possibly stronger negative effects of materialism, and how materialism and well-being may change across the life of these children.
